# Host heterogeneity in humoral bactericidal activity can be complement independent

**DOI:** 10.3389/fimmu.2024.1457174

**Published:** 2024-09-18

**Authors:** Ryuichiro Abe, Nikhil Ram-Mohan, Elizabeth Jordan Zudock, Shawna Lewis, Karen C. Carroll, Samuel Yang

**Affiliations:** ^1^ Department of Emergency Medicine, Stanford University School of Medicine, Palo Alto, CA, United States; ^2^ Laboratory of Bacterial Pathogenesis, International Research Center for Infectious Diseases, Research Institute for Microbial Diseases, Osaka University, Osaka, Japan; ^3^ Division of Medical Microbiology, Department of Pathology, Johns Hopkins University School of Medicine, Baltimore, MD, United States

**Keywords:** humoral bactericidal activity, plasma antibacterial activity, inter-individual heterogeneity, diagnosis of bacterial infection, bloodstream infection, humoral innate immunity, sepsis endotyping, trained immunity

## Abstract

**Background:**

Humoral bactericidal activity was first recognized nearly a century ago. However, the extent of inter-individual heterogeneity and the mechanisms underlying such heterogeneity beyond antibody or complement systems have not been well studied.

**Methods:**

The plasma bactericidal activity of five healthy volunteers were tested against 30 strains of Gram-negative uropathogens, Klebsiella pneumoniae and Escherichia coli, associated with bloodstream infections. IgG and IgM titers specific to K. pneumoniae strains KP13883 and KPB1 were measured by ELISA, and complement inhibitor was used to measure the contribution of complement-induced killing. Furthermore, MALDI-TOF mass spectrometry was conducted to determine the metabolomic components of plasma with bactericidal properties in 25 healthy individuals using Bayesian inference of Pearson correlation between peak intensity and colony counts of surviving bacteria.

**Results:**

Plasma bactericidal activity varied widely between individuals against various bacterial strains. While individual plasma with higher IgM titers specific to K. pneumoniae strain KP13883 showed more efficient killing of the strain, both IgM and IgG titers for K. pneumoniae strain KPB1 did not correlate well with the killing activity. Complement inhibition assays elucidated that the complement-mediated killing was not responsible for the inter-individual heterogeneity in either isolate. Subsequently, using MALDI-TOF mass spectrometry on plasmas of 25 healthy individuals, we identified several small molecules including gangliosides, pediocins, or saponins as candidates that showed negative correlation between peak intensities and colony forming units of the test bacteria.

**Conclusion:**

This is the first study to demonstrate the inter-individual heterogeneity of constitutive innate humoral bactericidal function quantitatively and that the heterogeneity can be independent of antibody or the complement system.

## Introduction

Bloodstream infections (BSIs), most often associated with bacterial pathogens, are a major cause of death worldwide with a one-month case-fatality rate as high as 13% (1). The establishment of bacterial BSIs involves complex interplays between pathogens and the host immune system. While asymptomatic bacteremia is frequently resolved in healthy individuals (2, 3), bacteremia in the setting of a maladaptive host response against the pathogen can lead to BSI that can rapidly escalate to life-threatening sepsis.

The innate arm of the immune system is the first line of host defense that utilizes constitutive and inducible mechanisms. Constitutive mechanisms, such as soluble mediators of the humoral immunity, have the advantage to provide direct killing upon initial pathogen contact (4, 5). By contrast, inducible mechanisms are activated only when stimulated, and can amplify signals for higher efficacy (5). While the cellular component has received significant attention mainly as part of inducible mechanisms, especially since the advent of single-cell technologies (4), the humoral constitutive arm has generally been considered to have only minor roles without specific, amplifiable killing functions in host defense, hence has not received much attention in immunological studies (5). However, recent evidence suggests that by reducing the levels of pathogen-associated molecular patterns through immediate antimicrobial effect, constitutive immune mechanisms can mitigate inducible cellular immune responses that could result in excessive inflammation and immunopathology (5–7).

Humoral immunity is a combination of various biological processes (8), including antibodies, the complement system, and other antimicrobial substances (9). In particular, the complement system is known to clear bacteria within minutes (10–13), and compromised complement system has been shown to increase susceptibility to bacterial infection (14, 15). Recognition of bacterial presence triggering the activation of the complement systems occurs via antibodies (classical pathway), mannose-binding lectin or ficolin (lectin pathway), or spontaneous and induced C3 hydrolysis (alternative pathway) (16). While the inter-individual variability in humoral bactericidal activity was first recognized nearly a century ago with suggestive clinical correlation with BSI (17), difference in antibody repertoires activating the complement system has been proposed as the primary mechanisms driving the heterogeneity of humoral immunity; and accordingly, serum antibody responses have been actively studied in vaccine development (17–20). However, the extent of inter-individual heterogeneity of constitutive humoral bactericidal activity remains unclear, and the soluble components in plasma other than antibody and the complement system have never been studied as potential contributors to the heterogeneity (21).

In this study, we analyzed the plasma bactericidal activity against BSI-related Gram-negative uropathogens since urinary tract infections are the most common cause of bacteremia (22, 23). Inter-individual heterogeneity of plasma bactericidal activity was investigated with respect to the contribution of antibodies and the complement system. Furthermore, we explored the inter-individual differences in plasma components using mass spectrometry and screened the candidate factors that may drive the inter-individual heterogeneity of humoral bactericidal activity.

## Materials and methods

### Bacterial strains and human plasmas

*Klebsiella pneumoniae* strain ATCC13883 (KP13883) and *Escherichia coli* strain ATCC 10789 (EC10789) were purchased for this study, and all the other isolates were sourced from the Johns Hopkins Hospital clinical microbiology laboratory. Seven *K. pneumoniae* and seven *E. coli* strains were isolated from the blood and urine of 28 patients. The results of antimicrobial susceptibility testing of the strains are shown in **Supplementary Table 1**. After informed consent, human plasma H1 to H25 was obtained from 25 healthy volunteers without any systemic infection, who had not taken any antibiotics in the two weeks prior to the blood draw. The demographics of each individual are provided in **Supplementary Table 2**. The study protocol (70759) was reviewed and approved by Stanford University’s Institutional Review Board. Blood was collected from healthy volunteers using BD Vacutainer Heparin tubes (BD, NJ, United States) and immediately centrifuged for 15 min at 4000 rpm at room temperature. The plasma was then aliquoted and preserved at –80°C. Plasma samples did not undergo multiple freeze-thaw cycles.

### Plasma bactericidal assay

Colonies of overnight bacterial cultures on Mueller Hinton II (MHII) agar plates were suspended in Dulbecco’s Phosphate-Buffered Saline (PBS) medium to be adjusted to a McFarland 1.0. Bacterial suspensions were further diluted 1:100 with PBS and 8 μL of this was mixed with 72 μL of plasma and 25 μL of buffer consisting of 80% Hank’s buffer and 20% Brain Heart Infusion (BHI) broth (final concentration, 2 × 10^5^ CFU/mL). After 3 h incubation at 37°C, 50 μL of the culture diluted to 1:10 and 1:100 with PBS was inoculated on MHII agar plate. Colonies were counted after overnight incubation at 37°C. All thirty strains were tested against the plasmas of five individuals (H1 – H5), and five strains that showed inter-individual heterogeneity in the assay were additionally tested against the remaining plasmas (H6 – H25) for subsequent mass spectrometry analysis. Proportions of overall killing between isolates from blood and urine were compared using Fisher’s exact test.

### Whole bacterial cell ELISA

Serum IgG and IgM levels of individuals (H1 - H5) in response to whole bacterial cells of *K. pneumoniae* KP13883 and KPB1, which showed strong inter-individual heterogeneity in the bactericidal assay, were determined by ELISA as previously described (24). KP13883 and KPB1 were incubated on MHII plates overnight, and colonies were picked and incubated in BHI broth at 37°C for 2 h, centrifuged, and washed with PBS at 4°C. One hundred microliters of suspension of bacterial cells adjusted to a McFarland 1.8 in PBS (5.4 × 10^8^ cell/mL) or PBS without bacteria were incubated in 96 well cell culture plate (Corning, NY, United States) at 4°C overnight to attach the bacterial whole cells on the wells. After washing by PBS with 0.05% Tween20, the wells were blocked with Blocking buffer (2× PBS with 0.2% Tween20 and 1% bovine serum albumin) for 2 h at room temperature. After the wells were washed, plasma or PBS (control) diluted 1:1000 with Dilution buffer (1× PBS with 0.1% Tween20 and 0.5% bovine serum albumin) was incubated in the wells for 2 h at room temperature. After washing, HRP-conjugated mouse anti-human IgG Fc (Invitrogen, MA, USA, Cat. Nos. 88-50550-88, RRID: AB_2574893) diluted 1:250 with Dilution buffer was incubated in the wells for 1h at room temperature. After washing, TMB substrate solution (Thermo Fisher Scientific, MA, United States) was incubated for 15 min, and the reaction was stopped by the addition of 2N H_2_SO_4_. The optical density (OD) at 450 nm and 570 nm was read on Synergy LX (BioTek, CA, United States). ELISA for IgM was conducted using HRP-conjugated mouse anti-human IgM heavy chain antibody (Invitrogen, Cat. Nos. 05-4920, AB_2532928) diluted at 1:1000 with Dilution buffer, and plasma diluted at 1:10 with Dilution buffer. All the assays were performed five times each.

### Complement inhibitor assay

KP13883 and KPB1 were cultured overnight on MHII agar plates, and the colonies were suspended in PBS medium to be adjusted at McFarland 1.0. Bacterial suspensions were further diluted with PBS medium at 1:100. 0.6 μL of Complement C5-IN-1 (MCE, NJ, United States) suspended in DMSO at 5 mM, or 0.6 μL of DMSO was added to 100 μL of plasma or PBS (25). Eight microliters of bacterial suspension were mixed with 72 μL of plasma or PBS with/without inhibitor and 20 μL of buffer consisting of 80% Hank’s buffer and 20% BHI broth. After 30 min or 3 h incubation at 37°C, 1:10 and 1:100 dilutions in PBS were prepared. Fifty microliters from each dilution and undiluted culture were inoculated on MHII agar plates. Following overnight culture at 37°C, the number of the colonies were counted. All the assays were performed five times each and the bactericidal activity of each plasma with and without the inhibitor was compared using Mann-Whitney U test.

The activity of complement inhibitor was confirmed by ELISA as previously described (25). Briefly, each plasma sample was diluted to 50% with PBS. 0.6 μL of C5-IN-1(5mM in DMSO) or DMSO was added to 100 μL of PBS or 50% plasma in PBS. One microliter of zymosan (100 μg/μL) was added to all the samples to initiate alternative pathway activation and incubated at 37°C for 40 min or 3 h. One hundred microliters of 0.05M EDTA were added to stop complement activation. Reaction products were absorbed in 96 well Nunc Maxisorp plate (Invitrogen) overnight at 4°C. The well was washed with PBS with 0.05% Tween20 and blocked by 250 μL Blocking buffer for 2 h at room temperature. After the well was washed, the complement membrane attack complex (MAC) was probed with mouse anti-human C9 neoepitope monoclonal antibody (aE11, Thermo Fisher Scientific) suspended in Dilution buffer (0.25 μg/μL). After washing, rat anti-mouse IgG2a HRP antibody (Invitrogen, MA, USA, Cat. Nos. 04-6220, RRID: AB_2532947) diluted 1:500 in Dilution buffer was used to detect MAC. After washing the wells, TMB substrate solution was incubated in the wells for 15 min, and the reaction was stopped by 2N H_2_SO_4_. The optical density (OD) at 450 nm and 570 nm was read on Synergy LX (BioTek, CA, United States). All the assays were performed five times each and the formation of MAC in each plasma with and without the inhibitor was compared using Mann-Whitney U test.

### Mass spectrometry analysis of plasmas

Plasma from 25 healthy volunteers was analyzed using MALDI-TOF mass spectrometry as previously described (26). Briefly, thawed plasma samples were diluted 1:10 in water. αlpha-cyanohydroxycinnamic acid was prepared as matrix solution by dissolving in acetonitrile/water 50/50 vol/vol containing 0.1% TFA at 10 mg/mL. Five μL of plasma and matrix were mixed and spotted in triplicate onto a stainless MALDI target plate (Bruker Daltonics, MA, United States). MALDI-TOF MS spectra were obtained on a Bruker microFlex MALDI-TOF MS (S/N 256969.00028) and the Bruker Daltonics FlexControl software (Vincent Coates Foundation Mass Spectrometry Laboratory, Stanford University). The parameters were as follows: linear mode, LP_ProtMix; laser power percentage was adjusted at 40% to obtain an arbitrary intensity of 10^3 – 10^4 and a flat baseline; mass range 2400–20000 Da; spectrum acquisition–random walk partial sample mode, results were the sum of at least 3 × 100 laser shots; calibration of the system was performed using Protein Calibration Standard I (Insulin [M+H]+ = 5,734.52, Cytochrome C [M+ 2H]2+ = 6,181.05, Myoglobin [M+ 2H]2+ = 8,476.66, Ubiquitin I [M+H]+ = 8,565.76, Cytochrome C [M+H]+ = 12,360.97, Myoglobin [M+H]+ = 16,952.31) (Bruker Daltonics). Data had been exported as an ASCII file from FlexAnalysis (Bruker Daltonics).

The raw mzXML spectra were analyzed using the R packages MALDIquant and MALDIquantForeign (27). Briefly, the imported raw spectra first underwent variance stabilization using the square root transformation and then the spectra were smoothed using the 21-point Savitzky-Golay-Filter with a half window size of 10. The baseline was then removed using the SNIP algorithm over 100 iterations. Next, the intensities were normalized using the Total-Ion-Calibration to remove small batch effects and to allow comparisons between spectra. The normalized spectra were then aligned using the lowess warping method with a half window size of 20, tolerance of 0.002, and SNR of 2. The technical replicates were then averaged and a final aligned spectrum with average intensities was used to detect peaks. An intensity matrix was then generated to perform Bayesian inference of Pearson correlation against the colony counts using the bayes.cor.test function from the BayesianFirstAid package (28). Peak m/z values were annotated by collecting the best hit against the Human Metabolome Database (https://hmdb.ca/).

## Results

### Inter-individual heterogeneity of bactericidal activity against different bacterial isolates

**Figure 1:**
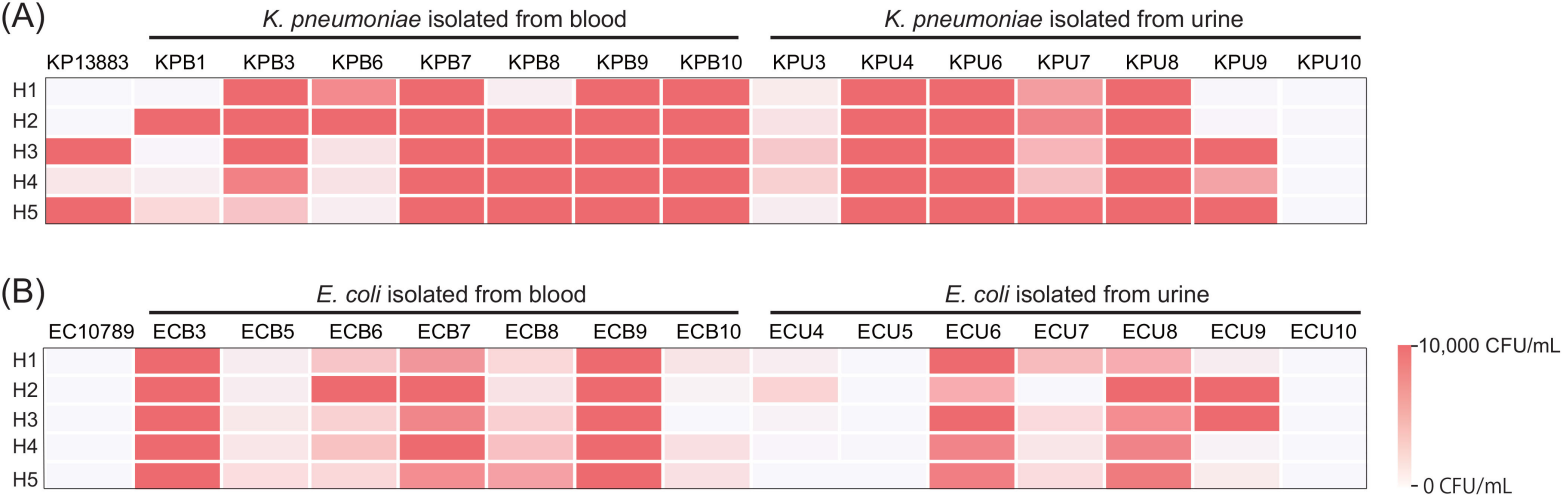
Extensive individual and isolate-level heterogeneity in bacterial clearance in plasma. Bactericidal activity of plasma from 5 healthy individuals against **(A)** *K*. *pneumoniae* and **(B)** *E*. *coli* isolates, 15 each (1 reference strain, 7 clinical isolates from blood, and 7 clinical isolates from urine), were measured by bacterial colony forming unit after 3h incubation with plasma. Bacteria were inoculated at an initial concentration of 200,000 CFU/mL. Survival greater than 10,000 CFU/mL was shown as 10,000 CFU/mL.

Bactericidal activity of plasma from five healthy volunteers against *K. pneumoniae* ATCC 13883 (KP13883) were measured (**Figure 1A**) and demonstrated high variability across individuals. Plasmas H1 and H2 showed complete killing, whereas plasmas H3 and H5 showed survival of over 10,000 CFU/ml. Plasma H4 also showed >99% killing, but less than plasmas H1 and H2. We further performed the same assay on fourteen *K. pneumoniae* isolated from either patients’ blood or urine. In addition to inter-individual heterogeneity, we observed inter-strain heterogeneity for the same individual. For example, within the same bacterial species, plasma H2 was highly bactericidal against KP13883 but least bactericidal against KPB1 and KPB6, while the exact opposite was true for plasma H3. A similar inter-individual and inter-strain heterogeneity was observed for *E. coli* (**Figure 1B**). Overall, 63.3% (19/30) of the strains showed congruity across the 5 plasmas in either achieving >99% killing (20%, 6/30) or not (43.3%, 13/30). The remainder 36.7% of the strains showcased inter-individual heterogeneity. For example, 10% (3/30) strains were killed by at least 99% by 4 plasmas; 13.3% (4/30) by 3 plasmas; and 6.7% (2/30) by both 2 and 1 plasma respectively. While some isolates from urine cultures were susceptible to all plasmas, isolates from blood cultures showed some resistance to at least one plasma sample. Across multiple replicates, only 2.9% (2/70) of instances with at least 99.9% killing of isolates from blood was observed in comparison to 25.7% (18/70) for isolates from urine (difference, 22.9%; 95% CI: 0.9% - 38.6%; p-value = 0.000147). Overall, isolates from urine were more susceptible to plasma bactericidal activity than those isolated from blood. Bacteria colonizing bloods might be more resistant against humoral components rather than bacteria colonizing urine due to their prior exposure to the bactericidal components in blood. All the strains were phenotypically distinct based on antimicrobial susceptibility testing (**Supplementary Table 1**) and plasma bactericidal assays (**Figure 1**), except for KPB9 and KPU6.

### Heterogeneity in bactericidal activity cannot only be attributed to antibody repertoires

**Figure 2:**
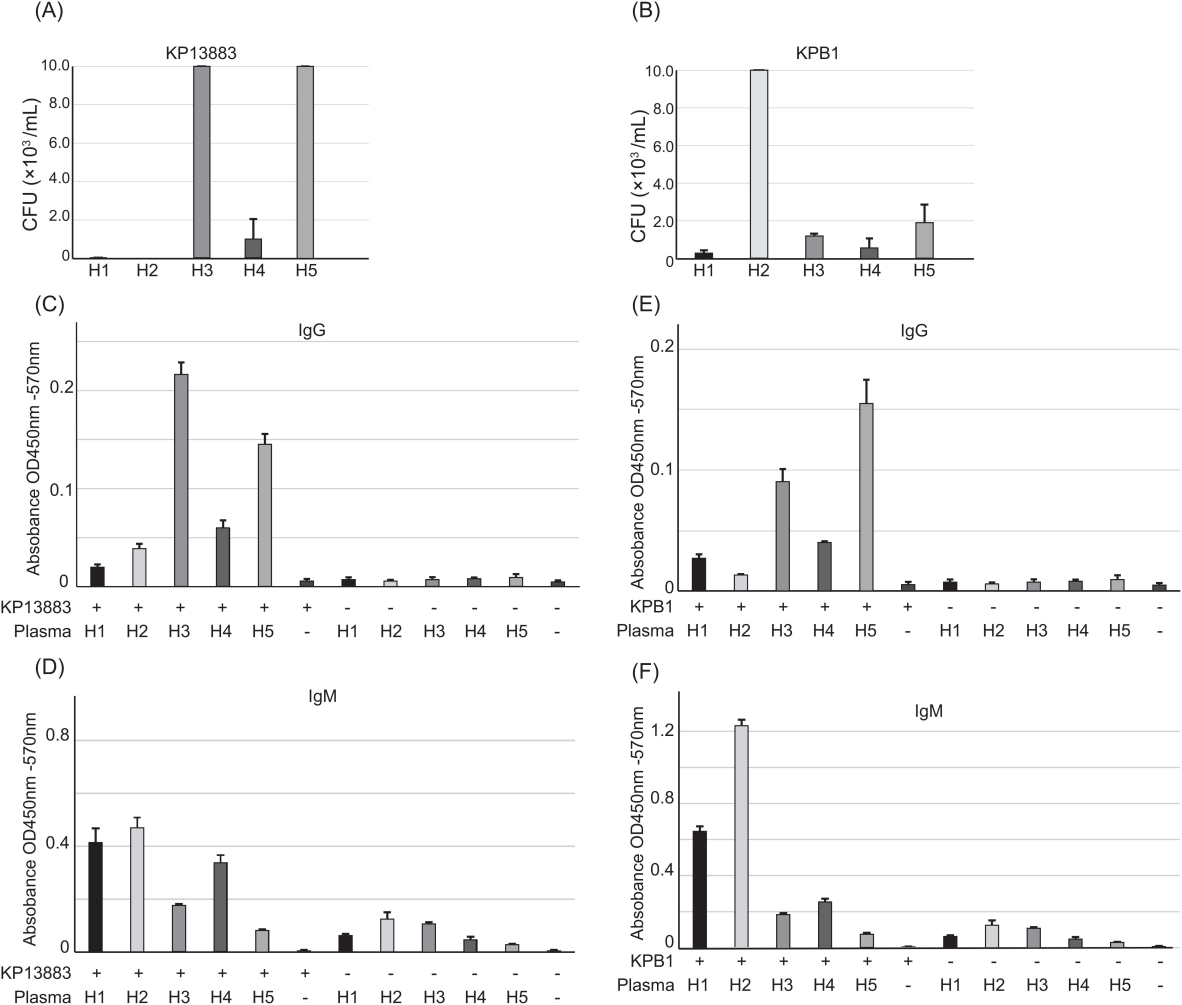
Individual antibody repertoire against KP13883 and KPB1. Plasma bactericidal activity against **(A)** KP13883 and **(B)** KPB1 were measured by counting bacterial colony forming unit after 3h incubation with plasmas. Inoculum concentration was 200,000 CFU/mL. **(C)** IgG titers and **(D)** IgM titers of individual plasmas against KP13883 were measured by bacterial whole cell ELISA. **(E)** IgG titers and **(F)** IgM titers of individual plasmas against KPB1 were measured by bacterial whole cell ELISA. Bars indicate the mean ± standard deviation, calculated from quintuple experiments.

We investigated the role of antibody repertoires in the observed heterogeneity of plasma bactericidal activity against KP13883 and KPB1 strains (**Figures 2A, B**). We performed a whole-cell ELISA against these two strains using five plasma samples to correlate the antibody titer against these strains with the extent of killing. We observed that plasmas H3 and H5, which did not kill KP13883, had concordant 1.9 - 5.7 times lower IgM titer and 2.9 – 10.9 times higher IgG titers than plasmas H1, H2, and H4 (**Figures 2C, D**). On the other hand, for KPB1, the IgG/IgM titers and bactericidal activity were not concordant. Plasmas H1 and H4 killed KPB1 more than H5 which had the highest IgG titer (**Figures 2B, E**), and plasma H2 had the highest IgM titer yet least killing of KPB1 (**Figures 2B, F**). These discrepancies suggested the lack of consistent correlation between the antibody titers and their bactericidal potency.

### Heterogeneity can be independent of the complement system

**Figure 3:**
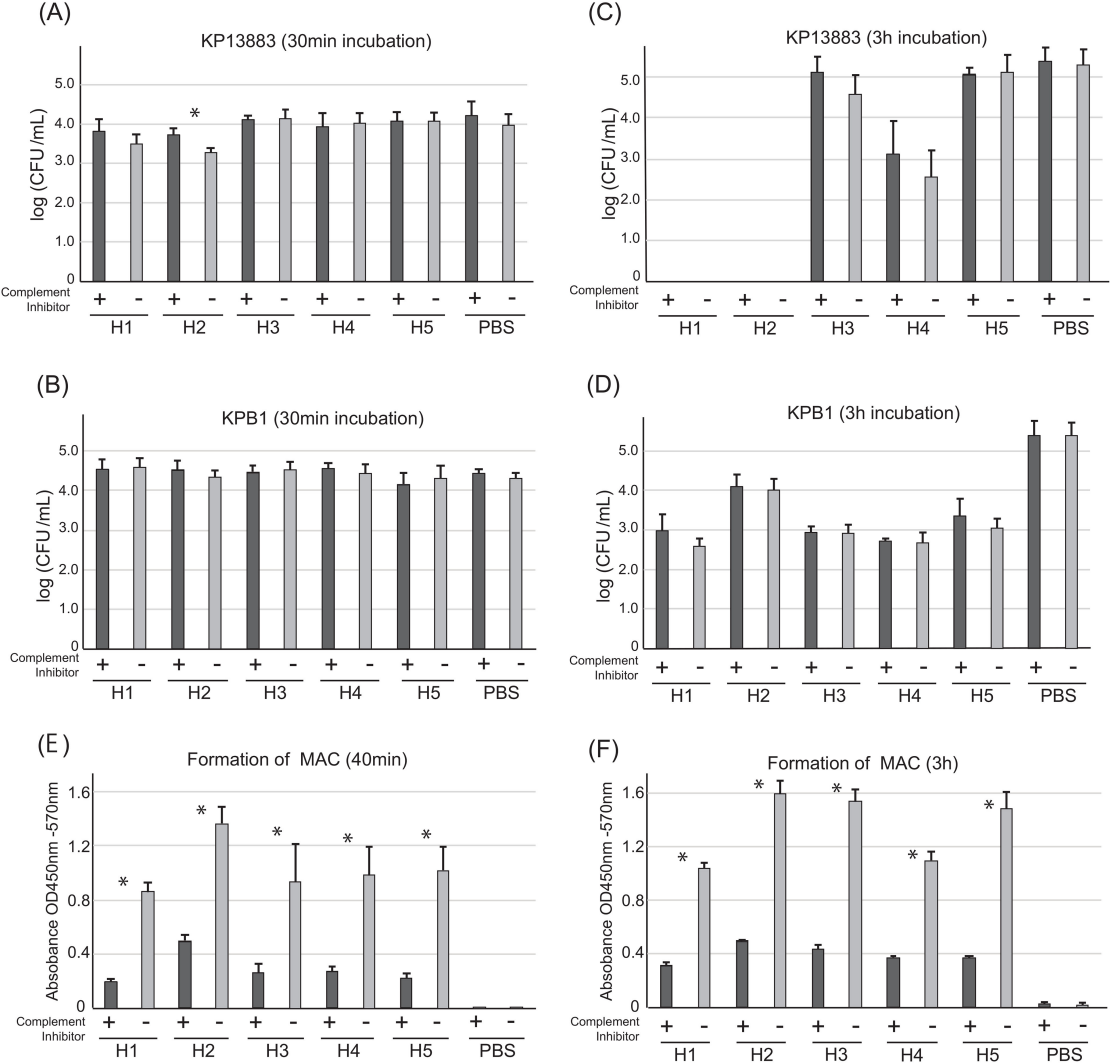
Contribution of complement system in plasma bactericidal activities against KP13883 and KPB1. Bactericidal activity against **(A)** KP13883 and **(B)** KPB1 after 30 min incubation with plasmas with or without C5 inhibitor was measured by CFU counting. Bactericidal activity against **(C)** KP13883 and **(D)** KPB1 after 3 h incubation with plasmas with or without C5 inhibitor was measured by CFU counting. **(E)** Inhibition to form membrane attack complex (MAC) after 40 min complement pathway activation. Inhibition of MAC formation by C5 inhibitor in each plasma was by ELISA using anti-human C9 antibody. **(F)** Inhibition of MAC formation by C5 inhibitor after 3 h complement pathway activation. Bars indicate the mean ± standard deviation, calculated from quintuple experiments. Statistical analysis was performed to compare each plasma with and without the complement inhibitor using Mann-Whitney U tests; *P < 0.05.

Because the humoral bactericidal activity of antibody is mediated via the complement system, we next performed a complement inhibitor assay to determine the contribution of the complement system to bactericidal activity using plasmas from the same 5 healthy volunteers. We assessed the change in bactericidal activity after using C5 inhibitor, which can inhibit all synthetic pathways including classical, lectin, and alternative pathways to form the membrane attack complex (MAC) (**Figure 3**). After 30 minutes of incubation in plasmas with and without C5 inhibitor, the number of KP13883 colonies was compared. Although complement-associated bactericidal activity occurs within minutes (13), we observed no significant differences with or without inhibitor except for plasma H2, which had the highest IgM titer against KP13883 (**Figure 3A**). On the other hand, against KPB1, all plasmas in the presence of C5 inhibitor did not result in significant decrease in bactericidal effect. (**Figure 3B**). Interestingly, the inter-individual variation in bactericidal effects was minimal at 30 minutes. Extending the incubation with plasma to 3 h resulted in marked inter-individual differences including complete killing of KP13883 in plasmas H1 and H2, but again, C5 inhibitor did not show any significant impact on bactericidal effect in all plasmas (**Figures 3C, D**). These results suggested the presence of strong bactericidal factors independent of the complement system involved in the observed differences in killing. Inhibition of MAC synthesis by C5 inhibitor in all plasmas at 30 min and 3 h was confirmed by ELISA using the anti-C9 antibody (**Figures 3E, F**). We also confirmed the presence of complement activity in all plasmas tested despite the presence of heparin, which has been reported to partially inhibit complement activity (29).

### Profiles of plasma components corresponding to inter-individual heterogeneity of bactericidal activity

**Figure 4:**
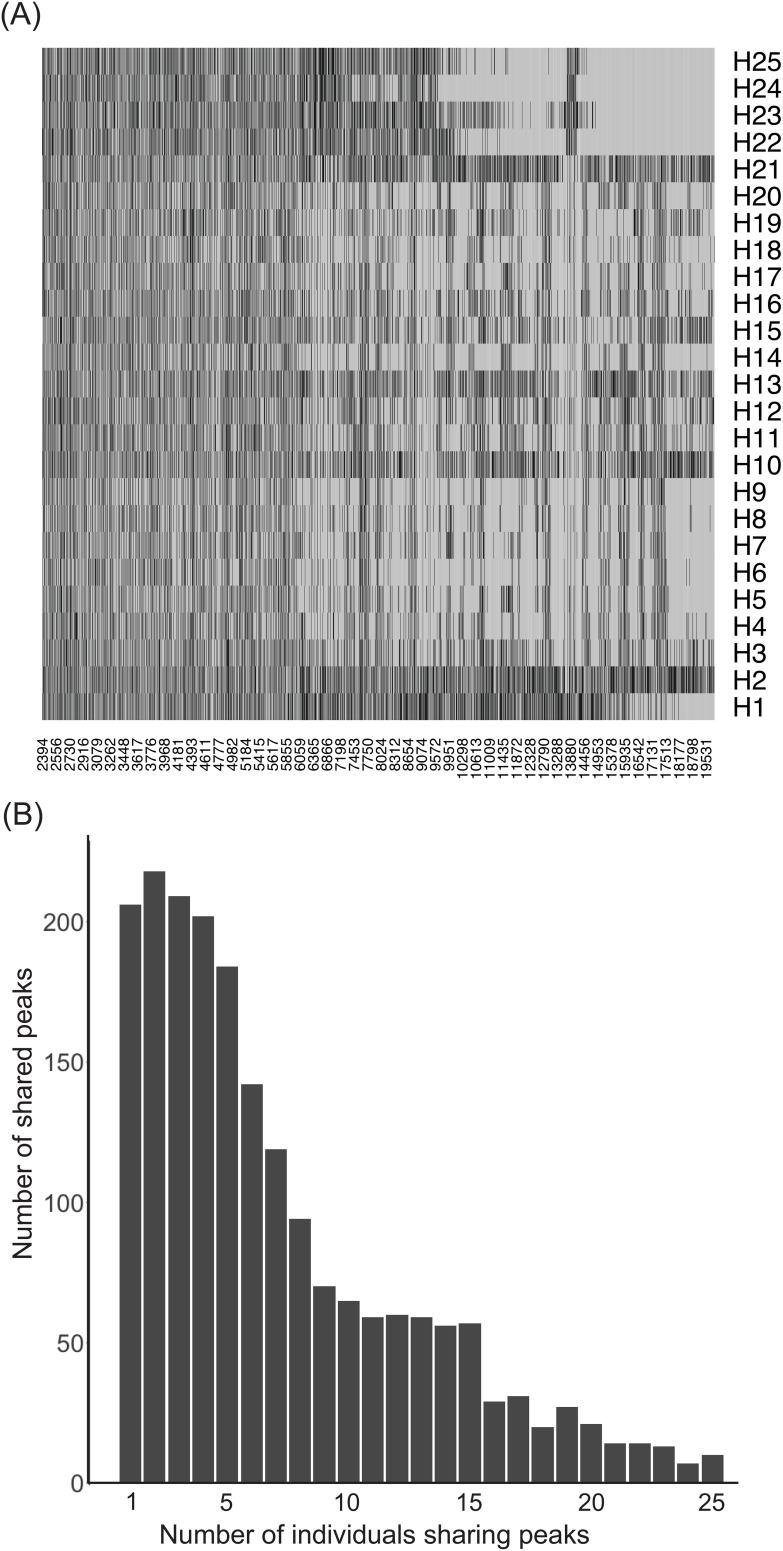
Heterogeneity in the detected MALDI peaks in the plasma of the 25 individuals. Extensive heterogeneity in the soluble metabolite peak pattern among healthy individuals. **(A)** A presence/absence heatmap depicting the heterogeneity in the called peaks (x axis) across individuals (y axis). Black regions represent called peaks. **(B)** Histogram of the number of peaks shared among individuals. Only 10/1986 detected peaks were shared among all 25 individuals. More than 50% of all peaks detected were shared by 5 or fewer individuals.

To explore plasma metabolome components contributing to inter-individual heterogeneity in bactericidal activity, we first performed mass spectrometry analysis on plasmas from 25 healthy individuals and then correlated plasma component profiles with their corresponding bactericidal activities. Of the peaks obtained, only peaks above background were analyzed. Across the 25 individuals, a total of 1,986 peaks were called median of 544 peaks with an interquartile range of 433 – 623 peaks. Interestingly, only 10 peaks (0.50%) were found to be common across all 25 MALDI-TOF MS spectra and 206 (~10.4%) were unique to different individuals (**Figure 4**). 51.3% of the peaks were shared among 5 or fewer individuals and only 3.9% of peaks were shared among at least 20/25 individuals suggesting extensive heterogeneity in the presence/absence of peaks across all individuals.

**Figure 5:**
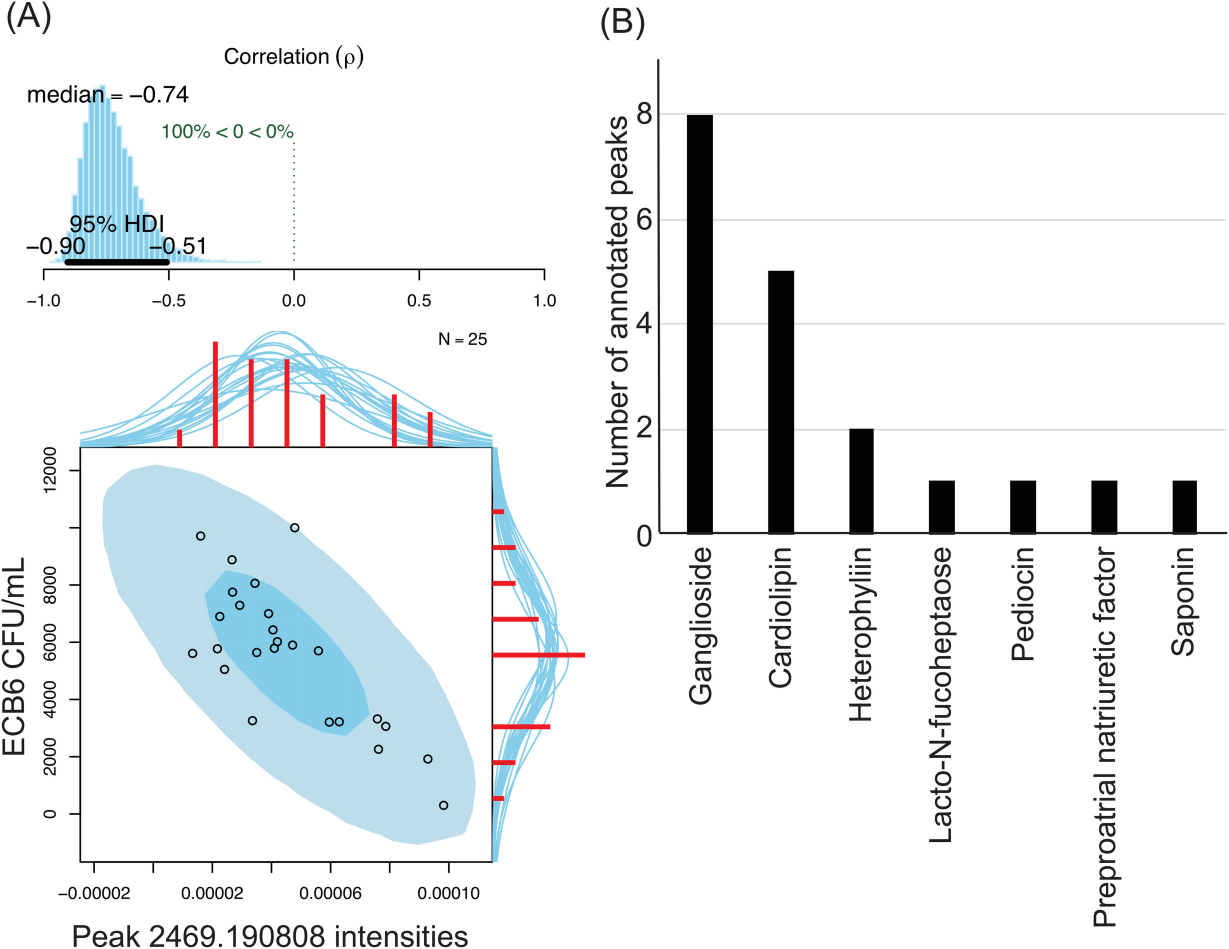
Soluble metabolites in plasma are negatively correlated with ECB6’s survivability in the individual. 333 peaks were negatively correlated with ECB6 and 4 were negatively correlated with ECU4. No negative correlation was observed against the tested KP strains. **(A)** Strong negative correlation between peak 2469.190808 intensities and ECB6 CFU/mL after treatment with plasma. Top: Posterior distribution for the Bayesian inference of Pearson correlation (-0.74) between the bacterial survivability and peak intensities with the 95% high density interval. Bottom: Original data plotted superimposed with the posterior predictive distributions. Red histograms represent the marginal distributions of the original data along with the marginal densities drawn from the posterior. **(B)** Annotatable peaks in plasma with negative correlation against ECB6. Peaks with a negative Bayesian inference of Pearson correlation of <-0.5 and the 95% high density interval falling <0 were selected for further annotation. m/z values were compared against those in the Human Metabolome Database and the best hit was collected. A distribution of the 19 annotatable peaks is represented with gangliosides being the most peaks with negative correlation against ECB6 followed by cardiolipins.

Next, we assessed the potential role of the detected soluble mediators in killing bacteria. Plasma from the 25 individuals were tested against 5 bacteria that showed strong heterogeneity of killing among 5 individuals – ECB6, ECU4, KP13883, KPB1, and KPB6, to assess their bactericidal activity using colony counts as described above (**Supplementary Figure 1**). For each of the 1,986 peaks, we determined the peak intensity in the MALDI-TOF MS spectrum for each individual and then performed Bayesian inference of Pearson correlation between the peak intensities and the colony counts for each healthy volunteer. The correlation between peak intensity in each individual and the ability of the individual to kill the bacteria suggests that the molecule represented by the peak potentially contributes to the bactericidal effect. No negative correlation, median rho of <= -0.5 with the limits of the 95% confidence interval being <0, was observed between peak intensities and colony counts for KP13883, KPB1, and KPB6. Only four peaks were moderately negatively correlated against ECU4 with median rho ranging from -0.50 - -0.55. For ECB6, 333 peaks were negatively correlated with the median rho ranging from -0.50 - -0.74, with the peak with m/z of 2469.190808 having the highest negative Bayesian inference of Pearson correlation (**Figure 5A**). The m/z values of the 337 peaks with significant negative correlation to the ECB6 were then annotated against the Human Metabolome Database (HMDB) and the closest hit was collected. Only 19 peaks were annotatable through the HMDB, and the majority were either gangliosides, including peak 2469.190808, or cardiolipins (**Figure 5B**). Other annotated molecules such as pediocins (bacteriocins), saponin, and heterophyllin were exposomes.

## Discussion

In this study, we demonstrated the extensive degree of inter-individual heterogeneity of plasma bactericidal activity that also varied across pathogens at the strain level. Contrary to the widely accepted notion that disparate antibody repertoires and complement pathways drive heterogeneity (17, 19, 30–34), we observed that antibody titers did not correlate well with the levels of killing and to firstly report that plasma bactericidal activity can be complement-independent. Mass spectrometry analysis revealed wide heterogeneity among individual plasma metabolome components profiles, which might be responsible for the heterogeneity of the plasma bactericidal activity. Bayesian inference of Pearson correlation analysis identified specific peaks correlated with bactericidal activity. Several annotatable small molecule families, including gangliosides, pediocins, or saponins, in plasma were likely associated with bactericidal activities. Given plasma bactericidal effect reflects the functional status of constitutive humoral immunity, a comprehensive exploration of the factors underlying the inter-individual heterogeneity in plasma bactericidal competence beyond antibody or complement systems may provide a new window to better understand the innate arm of the humoral immunity.

Despite the presumed dominant role of complement-mediated killing as part of humoral immunity with or without antibody activation, we were surprised by the lack of reduction in bactericidal potential by complement inhibition through all the pathways. Although it is well known that the complement system makes membrane attack complexes (MAC) pores to attack bacteria, the exact mechanism of MAC-mediated bacterial killing has been debated (35). The damage against the outer lipopolysaccharide (LPS)-containing membrane of bacteria caused by MAC is not by itself bactericidal unless secondary damage of inner, cytoplasmic membrane occurs (36). Furthermore, *K. pneumoniae* is known to evade from complement system by producing strain-specific extracellular capsules (37), and modifying the structures of LPS and outer membrane (38, 39). The bactericidal function of the complement system may be strain specific. In this study, our results showed that complement-mediated killing was ineffective against *K. pneumoniae* isolates KP13883 and KPB1. Despite complement inhibition by heparin or C5 inhibitor, bactericidal activities were still observed across individuals’ plasmas, some with strong effects, suggesting the presence of potent bactericidal factors independent of the complement system.

In recent years, an unexpected repertoire of various bactericidal substances in human blood has been reported (40). For instance, antimicrobial property has been additionally designated on known components, such as peptidoglycan recognition proteins and various chemokines (41, 42), and antimicrobial peptides, which may be useful in BSI management (43, 44), have been identified in human plasma components including lipoproteins (45–47). Although cell-free DNAs, RNAs, phageome or exposome in plasma may not be considered as part of the immunity, they also have antibacterial functions (48–52). However, the effects of all these factors on inter-individual differences in bactericidal activity has not yet been investigated. In the present study, we identified several annotatable small molecules in plasma associated with the inter-individual differences in killing of *E. coli*. While cardiolipin is a phospholipid found in the mitochondria of all mammalian tissues (53), it can be released into the circulation under pathological conditions (54) and elicit an antibody-independent activation of complement system (55), or inflammation as a mitochondrial damage-associated molecular patterns (DAMPs) (56). Gangliosides are common target cell surface receptors for bacterial entry into cells, but free gangliosides have been found in milk and serum (57),with direct antibacterial properties reported in gangliosides extracted from milk (58). Surprisingly, we also found several small molecules belonging to the “exposome”, or cumulative life-course exposures of the host, to be associated with plasma bactericidal effect. Pediocins (bacteriocins) are proteinaceous antimicrobials produced by bacteria, that function through membrane permeabilization (59), while saponin, which is isolated from the roots of legumes, has also been shown to have antibacterial property (60). Heterophyllin, which is also isolated from plants, has been studied as a potential anticancer drug (61, 62). It remains unclear if these aforementioned molecules have direct or indirect antimicrobial effects in plasma. Further studies are required to elucidate the mechanisms underlying the antimicrobial properties of these molecules and their potential impact on the inter-individual heterogeneity in humoral innate immunity. Interestingly, there is increasing evidence suggesting immunomodulatory effect of exposome on innate immune memory, so-called “trained immunity”, likely realized via epigenetic reprogramming of immune cells (63, 64). Furthermore, recent study has shown that the innate immune memory of invertebrates, known to lack adaptive immunity, can be achieved exclusively via humoral factors (65). As such, assuming immunity is conserved through evolution, it is conceivable that the observed inter-individual heterogeneity in plasma bactericidal effect may reflect each individual’s humoral innate “immunobiography” owning to exposure history and immune memory.

Meanwhile, invading bacterial pathogens have also evolved a myriad of mechanisms to elude host defenses. Host defenses generally target bacterial cell surface structures such as capsules, outer membrane proteins, and lipopolysaccharides, which can vary between species or even strains within the same species (66–70). Bacteria are known to produce strain-specific extracellular polysaccharide capsules that protect against humoral factors and express complement inhibitors. We observed similar findings from prior studies, which have reported higher serum-resistant strains isolated from blood cultures than from urine or feces (71–75). Bacteremia caused by serum-resistant *E. coli* has also been associated with shock and death (76). The various mechanisms responsible for the inter-individual heterogeneity of bactericidal activity may also be strain-specific, as we observed that the extent of bactericidal activity varied at the strain level (77). Considering the complexity of host-pathogen interplay and the diversity of virulence factors, testing the viability of infecting isolates from various clinical specimens in the same patient’s plasma may provide a holistic functional assessment of the immune response specific to the suspected pathogens (21). Given the current efforts to classify sepsis patients into endotypes based on underlying immunobiology to better account for the heterogeneity in sepsis syndrome, an immune functional assay like the plasma bactericidal test may help define a distinct sepsis endotype, enabling personalized treatment. With the potential use of advanced technologies such as microfluidics for rapid microbial isolation from extravascular infections, the test may be performed prior to initiating antimicrobial therapy for more timely and reliable results in the future.

Humoral immunity has been implicated in the severity of sepsis, but there has been a lack of understanding on the details of inter-individual heterogeneity and its clinical implications in the context of BSI and sepsis. Despite of the small sample size, our study revealed the inter-individual heterogeneity of constitutive humoral bactericidal activity can be contributed by factors beyond the antibody or complement systems. Although our mass spectrometry analysis suggested the potential contribution of several molecules for this heterogeneity, it is difficult to accurately annotate and quantify using MALDI-TOF MS alone. Further analysis with better fractionation of the plasma components is needed to determine the molecules contributing to the inter-individual differences of bactericidal activity. Grouping the samples based on the backgrounds of their hosts may facilitate the identification of the molecules by simplifying the variation of plasma components. The combination of omics-based technologies may be used to elucidate the role of humoral innate immunity in trained immunity. Moreover, large-scale clinical studies enrolling both patients with or without bacteremia are needed to clarify the clinical relevance of inter-individual heterogeneity in humoral bactericidal activity. Modern technologies for rapid isolation of infecting bacteria directly from clinical specimens may allow immune-functional testing of pathogen-specific inter-individual humoral bactericidal competence for novel sepsis endotyping. While the cellular immunity has received the significant attention in the frontline research of immunology, application of the latest technologies to the study of humoral immunity may provide a new approach to the clinical testing that will aid in the personalized management of bloodstream infections and sepsis.

## Data Availability

The original contributions presented in the study are included in the article/**Supplementary Material**. Further inquiries can be directed to the corresponding author.
