# Supplementary material for: Host heterogeneity in humoral bactericidal activity can be complement independent
Source: Front Immunol. 2024 Sep 18;15:1457174. doi: 10.3389/fimmu.2024.1457174 (PMC11445025; doi:10.3389/fimmu.2024.1457174)
Supplement: Supplementary file 1 [file DataSheet1.pdf]

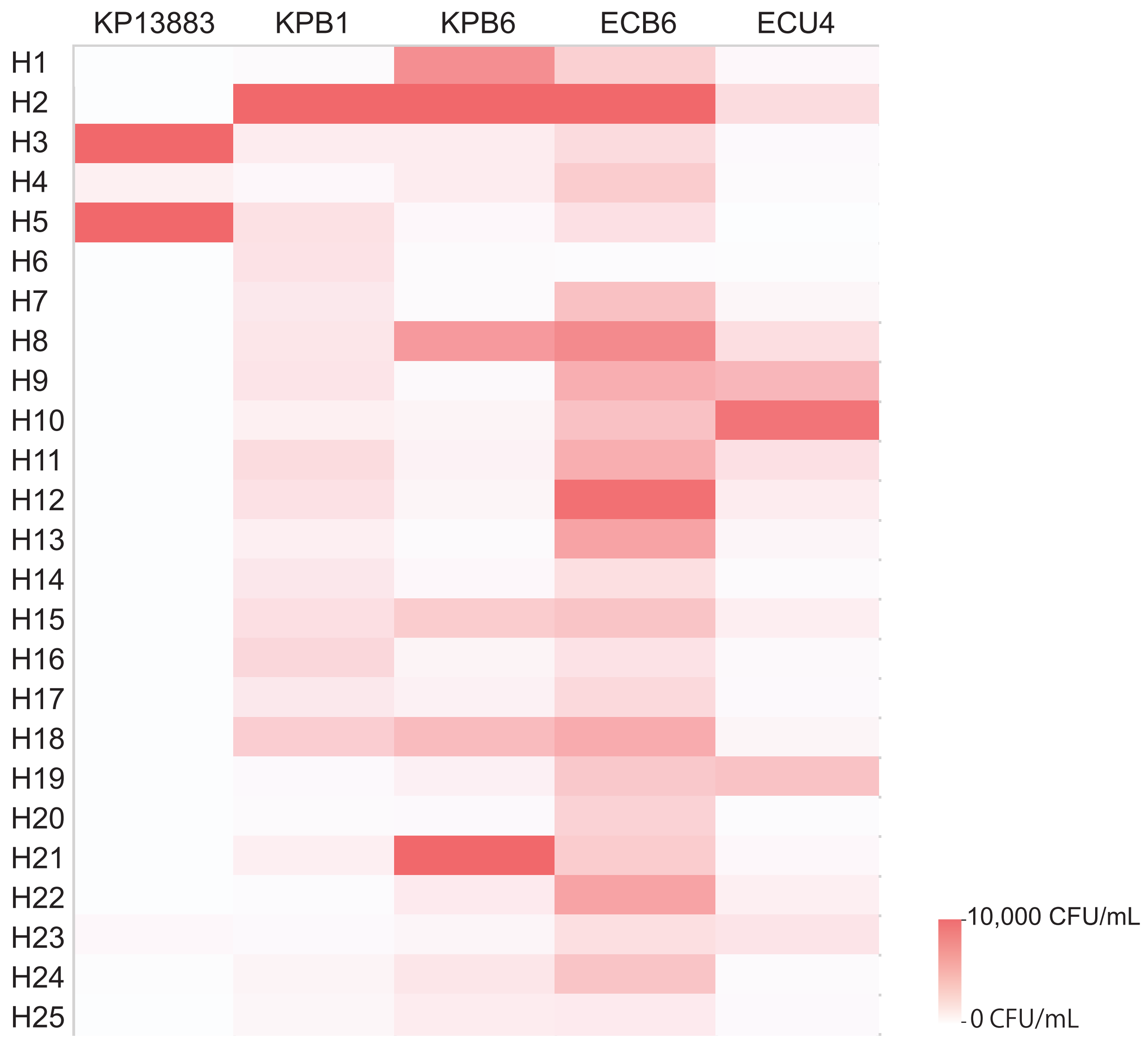

Fig S1. Inter-individual heterogeneity of plasma bactericidal activities.
Surviving bacterial colony forming units were counted after 3h incubation in 25 volunteers' plasmas. Inoculum concentration was 200,000 CFU/mL.
