# Supplementary material for: Host heterogeneity in humoral bactericidal activity can be complement independent
Source: Front Immunol. 2024 Sep 18;15:1457174. doi: 10.3389/fimmu.2024.1457174 (PMC11445025; doi:10.3389/fimmu.2024.1457174)
Supplement: Supplementary file 2 [file DataSheet2.pdf]

Table S1. Antimicrobial susceptibility profiling with MIC (mg/L)

Strain	Amikacin	Gentamicin	Ciprofloxacin	Ampicillin	Ceftriaxone	Ceftazidime	Meropenem	Cefazolin	Tetracycline
KP13883	<=8 S	<=2 S	<=0.25 S	>16 R	<=1 S	<=2 S	<=0.5 S	<=1 S	8 I
KPB1	<=8 S	<=2 S	<=0.25 S	>16 R	<=1 S	<=2 S	<=0.5 S	2 S	<=2 S
KPB3	<=8 S	<=2 S	<=0.25 S	>16 R	<=1 S	<=2 S	<=0.5 S	<=1 S	>8 R
KPB6	<=8 S	<=2 S	0.5 I	16 R	<=1 S	<=2 S	<=0.5 S	4 I	4 S
KPB7	<=8 S	<=2 S	1 R	>16 R	>32 R	8 I	<=0.5 S	>16 R	>8 R
KPB8	<=8 S	<=2 S	1 R	16 R	<=1 S	<=2 S	<=0.5 S	2 S	>8 R
KPB9	<=8 S	<=2 S	<=0.25 S	>16 R	<=1 S	<=2 S	<=0.5 S	2 S	>8 R
KPB10	<=8 S	<=2 S	<=0.25 S	>16 R	<=1 S	<=2 S	<=0.5 S	2 S	4 S
KPU3	<=8 S	<=2 S	<=0.25 S	16 R	<=1 S	<=2 S	<=0.5 S	<=1 S	<=2 S
KPU4	<=8 S	<=2 S	<=0.25 S	>16 R	<=1 S	<=2 S	<=0.5 S	16 S	<=2 S
KPU6	<=8 S	<=2 S	<=0.25 S	>16 R	<=1 S	<=2 S	<=0.5 S	2 S	>8 R
KPU7	<=8 S	<=2 S	<=0.25 S	>16 R	<=1 S	<=2 S	<=0.5 S	<=1 S	<=2 S
KPU8	<=8 S	<=2 S	<=0.25 S	16 R	<=1 S	<=2 S	<=0.5 S	<=1 S	<=2 S
KPU9	<=8 S	<=2 S	>2 R	>16 R	<=1 S	<=2 S	<=0.5 S	4 S	>8 R
KPU10	<=8 S	<=2 S	<=0.25 S	>16 R	32 R	>16 R	<=0.5 S	>16 R	<=2 S
EC10789	<=8 S	<=2 S	<=0.25 S	<=4 S	<=1 S	<=2 S	<=0.5 S		
ECB3	<=8 S	<=2 S	0.5 I	>16 R	>32 R	16 R	<=0.5 S		
ECB5	<=8 S	>8 R	>2 R	>16 R	>32 R	>16 R	<=0.5 S		
ECB6	<=8 S	<=2 S	<=0.25 S	<=4 S	<=1 S	<=2 S	<=0.5 S		
ECB7	<=8 S	<=2 S	<=0.25 S	<=4 S	<=1 S	<=2 S	<=0.5 S		
ECB8	<=8 S	<=2 S	<=0.25 S	>16 R	<=1 S	<=2 S	<=0.5 S		
ECB9	<=8 S	<=2 S	<=0.25 S	<=4 S	<=1 S	<=2 S	<=0.5 S		
ECB10	<=8 S	<=2 S	<=0.25 S	<=4 S	<=1 S	<=2 S	<=0.5 S		
ECU4	<=8 S	<=2 S	<=0.25 S	>16 R	<=1 S	<=2 S	<=0.5 S		
ECU5	<=8 S	<=2 S	>2 R	>16 R	<=1 S	<=2 S	<=0.5 S		
ECU6	<=8 S	<=2 S	<=0.25 S	<=4 S	<=1 S	<=2 S	<=0.5 S		
ECU7	<=8 S	<=2 S	<=0.25 S	<=4 S	<=1 S	<=2 S	<=0.5 S		
ECU8	<=8 S	4 S	<=0.25 S	>16 R	>32 R	8 I	<=0.5 S		
ECU9	<=8 S	<=2 S	0.5 I	>16 R	<=1 S	<=2 S	<=0.5 S		
ECU10	<=8 S	<=2 S	<=0.25 S	>16 R	<=1 S	<=2 S	<=0.5 S		

MICs were determined by the microdilution method based on CLSI M200-S26.
The antimicrobial susceptibility was indicated as follows; R, resistance; I, intermediate; S, susceptible.
