# Supplementary material for: Host heterogeneity in humoral bactericidal activity can be complement independent
Source: Front Immunol. 2024 Sep 18;15:1457174. doi: 10.3389/fimmu.2024.1457174 (PMC11445025; doi:10.3389/fimmu.2024.1457174)
Supplement: Supplementary file 3 [file DataSheet3.pdf]

Table S2. Characteristics of healthy volunteers (n=25)

Characteristic	Value
Age (years)	
Mean ± SD	37.32 ± 9.9
Range	22 - 70
Sex	
Female	12
Male	13
Race	
Asian	18
Caucasian	7

	Age	Sex	Nationality	Race
H1	30s	M	Japan	Asian
H2	20s	F	United states	Caucasian
H3	50s	M	Taiwan	Asian
H4	30s	M	India	Asian
H5	30s	F	Indonesia	Asian
H6	40s	M	Japan	Asian
H7	30s	M	Japan	Asian
H8	40s	M	Japan	Asian
H9	50s	M	Germany	Caucasian
H10	30s	M	Slovakia	Caucasian
H11	30s	M	Japan	Asian
H12	30s	F	United states	Asian
H13	30s	F	Japan	Asian
H14	40s	M	Japan	Asian
H15	30s	M	Japan	Asian
H16	30s	M	Japan	Asian
H17	30s	M	France	Caucasian
H18	20s	F	Netherland	Caucasian
H19	20s	F	United states	Caucasian
H20	20s	F	China	Asian
H21	30s	F	Japan	Asian
H22	70s	F	Japan	Asian
H23	30s	F	China	Asian
H24	20s	F	China	Asian
H25	20s	F	United states	Caucasian
